# The Paradox of Support: Mechanical Ventilation-Induced Hypoxemia Through a Patent Foramen Ovale

**DOI:** 10.7759/cureus.111033

**Published:** 2026-06-17

**Authors:** Mohamad A Soudan, Muhammad Awais, Osama Hallak, Steven J Filby

**Affiliations:** 1 Internal Medicine, University Hospitals Cleveland Medical Center, Cleveland, USA; 2 Cardiovascular Medicine, Hayatabad Medical Complex Peshawar, Peshawar, PAK; 3 Interventional Cardiovascular Medicine, Case Western Reserve University, Cleveland, USA; 4 Cardiology, University Hospitals Cleveland Medical Center, Cleveland, USA

**Keywords:** mechanical ventilation, patent forman ovale (pfo), pulmonary pressure, severe hypoxemia, ventriculo-atrial shunt

## Abstract

Patent foramen ovale (PFO) is common and usually harmless, but can cause significant positional and refractory hypoxemia in situations when there is a significant right-to-left shunt across it, and this phenomenon often goes unnoticed, especially in patients on ventilators, in whom it can be fatal. Platypnea-orthodeoxia syndrome (POS) is a condition characterized by positional dyspnea (platypnea) and hypoxemia (orthodeoxia), usually associated with right-to-left interatrial shunting across a PFO or an atrial septal defect (ASD). The positional variation is believed to be due to interatrial stretch, facilitating right-to-left shunt across PFO.

We report a 79-year-old woman with longstanding dyspnea, obstructive sleep apnea, and a remote history of pulmonary embolism on chronic anticoagulation who was admitted with worsening hypoxia that did not improve with bronchodilators, steroids, or oxygen therapy. Her workup, including chest computed tomography (CT) angiography, was unremarkable. Clinically, she continued to worsen, leading to severe respiratory failure requiring intubation with high oxygen and positive end-expiratory pressure (PEEP) requirements. Cardiac workup revealed a large PFO with right-to-left shunting, and its subsequent closure with an Amplatzer device led to rapid improvement in oxygen levels and prompt extubation.

PFO should be considered in anyone with positional dyspnea and hypoxemia or persistent refractory hypoxemia despite being on adequate ventilatory support. Early closure can be highly effective in selected patients.

## Introduction

A patent foramen ovale (PFO) is a persistent flap-like opening between the right and left atria due to incomplete postnatal closure of the foramen ovale and can lead to right-to-left shunt when right atrial pressure exceeds left atrial pressure. Its prevalence is 20 to 30% in the general population [1], and conditions that raise right atrial pressure, like coughing, valsalva, pulmonary embolism, right ventricular failure, or positive pressure ventilation, can lead to right-to-left shunting of deoxygenated blood. 

Patients with PFO are typically asymptomatic, and the defect goes undetected; however, in certain conditions, a significant shunt across it can lead to fatal conditions like unexplained or cryptogenic stroke, positional dyspnea, and hypoxemia or refractory hypoxemia despite adequate oxygen therapy, which should prompt evaluation for PFO. 

Platypnea-orthodeoxia syndrome (POS) is a rare condition where a patient develops shortness of breath and arterial desaturation when sitting or standing, with improvement while lying down. Its mechanism is position-dependent right-to-left shunting. In the upright posture, anatomical and hemodynamic changes like stretching of the interatrial septum, increased venous return to the right atrium, gravitational redirection of blood flow, or reduced left atrial filling promote opening of the PFO flap, allowing deoxygenated blood to bypass the lungs and enter systemic circulation [2]. POS is characterized by dyspnea and hypoxemia when upright, defined as a drop in PaO2 >4 mm Hg or SaO2 >5% [3].

In this report, we describe an elderly woman with chronic incompletely explained refractory dyspnea with positional variations who presented with acute hypoxic respiratory failure requiring intubation and escalating ventilator settings. A large PFO with right-to-left shunt was ultimately identified to be the cause of refractory hypoxemia and closed with immediate improvement in oxygenation. 

## Case presentation

Our patient is a 79-year-old woman with a history of chronic exertional dyspnea, obstructive sleep apnea, hypertension, glaucoma, idiopathic peripheral neuropathy, and a remote provoked small subsegmental pulmonary embolism (on apixaban), and mild obstructive airways disease. Her clinical response to these therapies remained suboptimal, with persistent hypoxemia. Moreover, she noticed variation in symptoms with postural changes. 

She presented to an outside hospital with worsening dyspnea, wheezing, and refractory hypoxemia. Imaging studies, including chest radiography and computed tomography (CT), pulmonary angiography, were unremarkable. She was empirically treated for asthma exacerbation with steroids, bronchodilators, diuresis, and antibiotics. Her oxygen saturations remained in the 70s despite maximal therapy, with slight improvement when supine. 

Her course progressed to the point of refractory hypoxemia despite noninvasive support. She was intubated for acute hypoxemic respiratory failure requiring fraction of inspired oxygen (FiO2) 1.0 and positive end-expiratory pressure (PEEP) 20 cm H2O and was transferred to our tertiary intensive care unit for further management. Despite maximal efforts, her oxygen saturation remained in the 70s. Right and left heart catheterization findings are summarised in Table 1, and oxygen saturation measurements from the shunt run are presented in Table 2.

**Table 1:** Hemodynamic findings on right and left heart catheterization

Parameter	Value
Right atrial pressure	12 mmHg
Right ventricular pressure	26/8 mmHg
Pulmonary artery pressure	24/5 mmHg
Pulmonary capillary wedge pressure	12 mmHg
Left ventricular end diastolic pressure	8 mmHg
Cardiac output	5.5 L/min
Cardiac index	2.9 L/min/m2

**Table 2:** Oxygen saturation measurements from shunt run No significant step up in oxygen saturation was observed.

Site	Oxygen saturation (%)
Inferior vena cava	70
Superior vena cava	69
Right atrium	70
Pulmonary artery	70
Left atrium	96
Left ventricle	92

**Figure 1:**
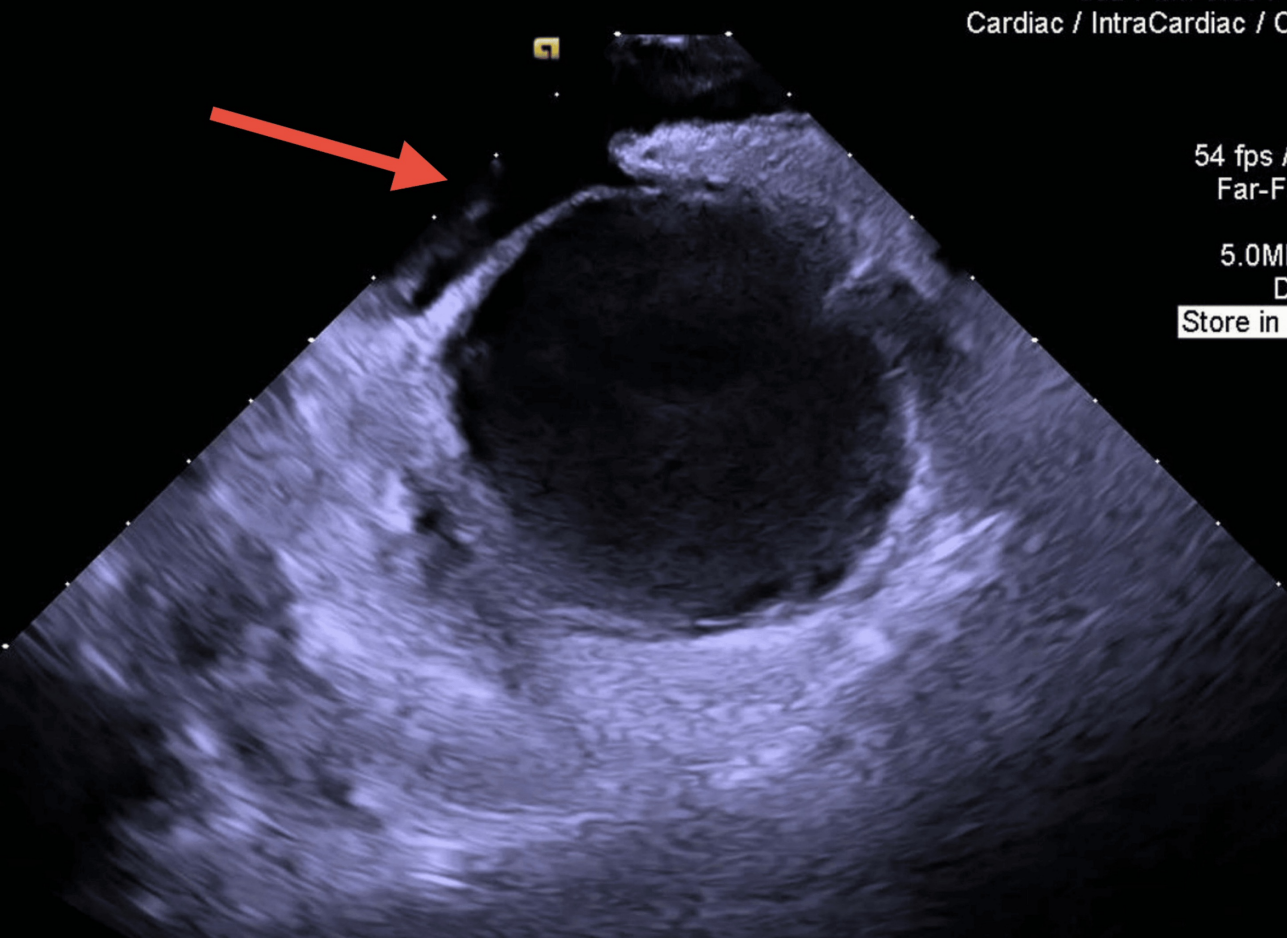
Intracardiac echocardiography (ICE) image showing a patent foramen ovale The septum primum flap is seen separating from the septum secundum, creating a pressure-dependent right to left shunt pathway.

**Figure 2:**
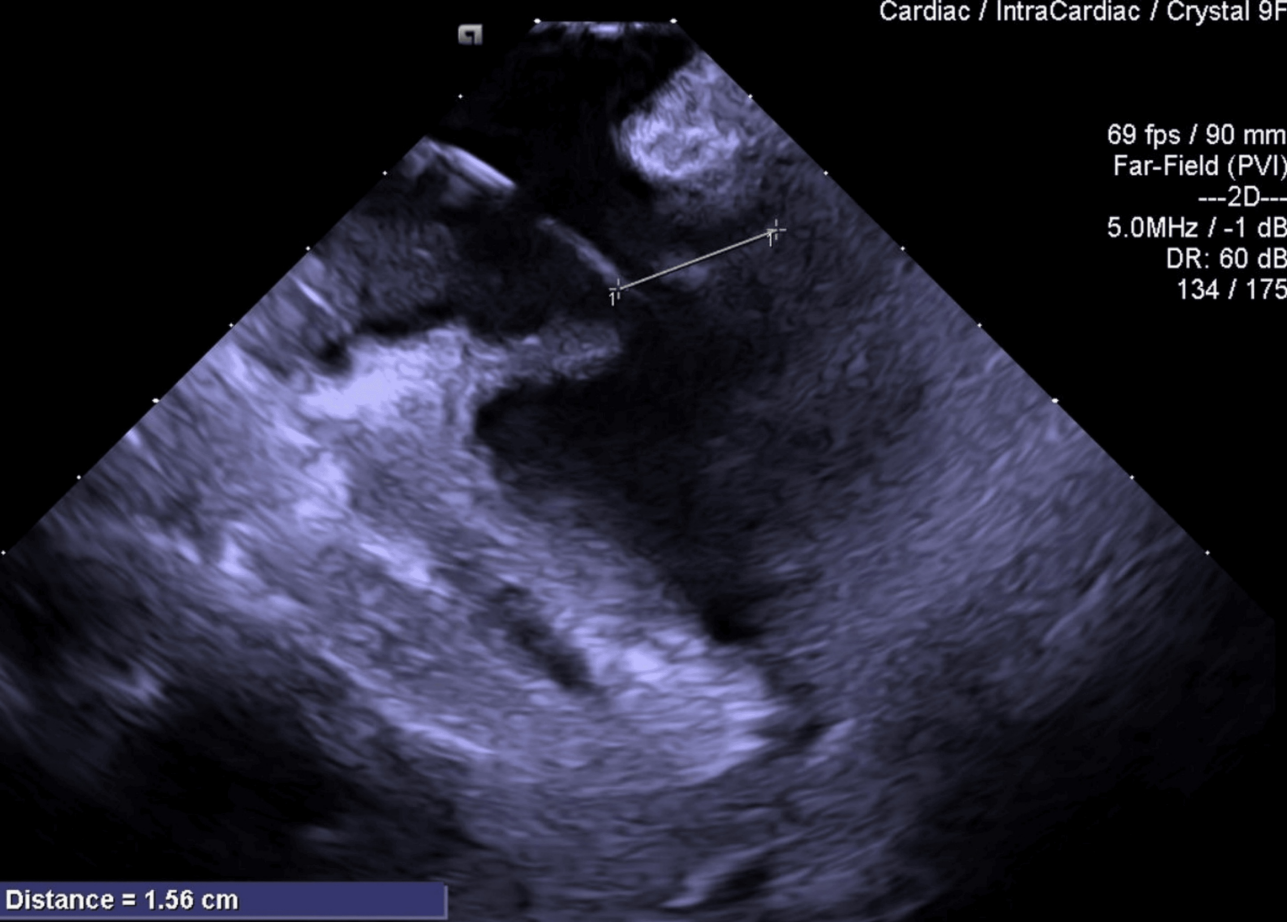
Measurement of patent foramen ovale (PFO) tunnel obtained using intracardiac echocardiography (ICE) Tunnel length measured to be 1.56cm, an elongated morphology associated with significant positional shunting and symptomatic platypnea-orthodeoxia syndrome (POS).

However, intracardiac echocardiography (ICE) showed a large patent foramen ovale (PFO) with bidirectional flow and prominent right-to-left shunting (Figures 1, 2).

**Figure 3:**
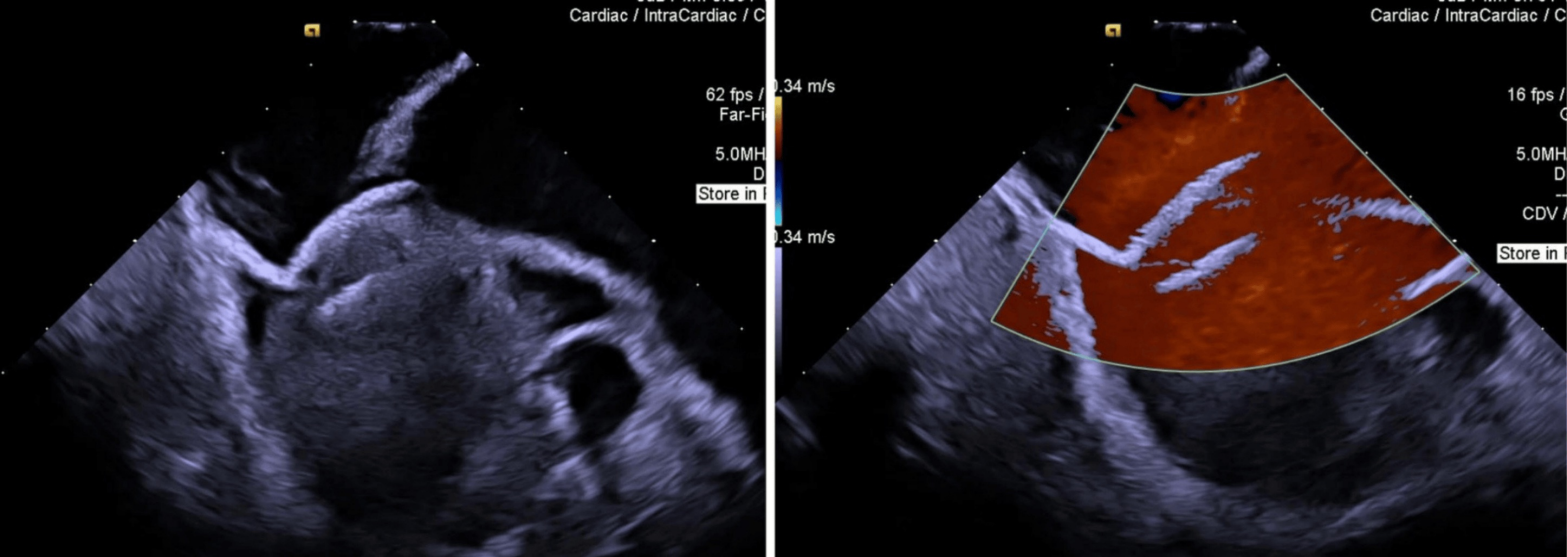
Intracardiac echocardiography (ICE) guided patent foramen ovale (PFO) closure The left panel demonstrates the PFO closure device during deployment. The right panel shows post deployment colour Doppler with complete abolition of the right to left interatrial shunt and stable device positioning.

After a multidisciplinary team discussion, it was concluded that shunting across the PFO could be the main contributor to hypoxemia, and the decision was made to close it. She underwent successful placement of a 30 mm Amplatzer Talisman septal occluder device under intracardiac echocardiographic and fluoroscopic guidance (Figure 3).

She had an immediate improvement in her ventilator requirements from FiO2 1.0 and PEEP 12 cm H2O to FiO2 0.40 and PEEP 5 cm H2O without recurrent desaturation. Attempted extubation after the procedure was associated with inadvertent oropharyngeal bleeding. The patient was successfully extubated on the following day and discharged on antiplatelet therapy with outpatient follow-up. 

## Discussion

Detecting platypnea‐orthodeoxia syndrome (POS) can be challenging as the diagnosis often presents with inconspicuous signs; however, one must have a suspicion for patent foramen ovale (PFO) whenever hypoxemia is out of proportion to the clinical scenario, despite adequate treatment, or when patients exhibit positional variations in oxygen saturation. An oxygen saturation that decreases by more than 5% in the upright position, with an improvement when assuming the supine position, should raise suspicion of POS. Dyspnea experienced in the upright position that disappears when assuming a lying position, along with orthodeoxia (sPO2 < 90% or pO2 < 60 mmHg in the upright position, normalization in the lying position), may also serve as an additional indicator. Heightened suspicion should arise if hypoxemia fails to improve with 100% fraction of inspired oxygen (FiO2), suggesting the presence of a clinically significant right-to-left shunt. 

This case highlights several important diagnostic caveats in the evaluation of refractory hypoxemia despite appropriate therapies. As an outpatient, her obstructive sleep apnea was not optimally controlled with the use of continuous positive airway pressure, and this observation should raise consideration of a PFO or intracardiac shunt in the appropriate clinical context. Patients with severe obstructive sleep apnea have a higher prevalence of PFO with large shunts compared with control subjects [4]. During obstructive sleep apnea, the strong inspiratory effort against a closed airway creates very negative thoracic pressure, which facilitates increased venous return and, in turn, raised right atrial pressure and more right-to-left shunt across PFO. Besides hypoxemia, PFO in obstructive sleep apnea patients also carries a potential risk for cryptogenic stroke [5]. 

She also had treatment for asthma and chronic obstructive pulmonary disease (COPD). Studies have suggested that a PFO may be one of the main factors contributing to hypoxemia in patients with COPD, and that refractory hypoxemic COPD patients should be tested for PFO [6].

This patient ultimately needed assisted ventilation for worsening refractory hypoxemia, and while being on a ventilator, with maximal support, refractory hypoxemia persisted, which raised the suspicion for an alternate diagnosis. In ventilated patients, the diagnosis of POS is particularly challenging because the positional testing required for recognizing POS is often impossible. Sedation, supine positioning, and the confounding effects of ventilator pressures can mask the typical orthodeoxia seen when a patient sits upright. Several reports have described severe hypoxemia in mechanically ventilated patients where a PFO, previously silent, becomes a major source of right-to-left shunt. In a prospective study of ventilated intensive care unit patients, Vavlitou et al. demonstrated that elevated plateau pressures above 26 cm H₂O and acute right ventricular dilation significantly increased the likelihood of PFO opening [7]. 

In this case, intracardiac echocardiography (ICE) and cardiac catheterization revealed a significant right-to-left shunt across PFO, and its device closure ultimately led to symptomatic improvement. PFO closure reduces apnea hypopnea events, oxygen desaturation index, systemic and pulmonary blood pressure [7]. 

This patient also had a history of subsegmental pulmonary embolism, and studies have shown higher mortality and stroke risks in patients with pulmonary embolism having PFO [8]. 

Besides ventilatory mechanics, altered chest wall, unilateral diaphragmatic paralysis, postsurgical chest distortion, hyper-inflated lung, mediastinal shift, aortic root dilation, kyphoscoliosis, and post-pneumonectomy anatomy can stretch the atrial septum or change venous inflow patterns, allowing right-to-left shunt even in the absence of elevated right-sided pressures leading to POS. 

Atrial compliance also influences shunt behavior. A stiff or noncompliant left atrium due to age, diastolic dysfunction, or external compression reduces the normal dominance of left atrial pressure. Under stress, transient rises in right atrial pressure caused by coughing, ventilator cycling, increased work of breathing, or Valsalva-like effort can easily overcome a less compliant left atrium, opening the PFO flap and enabling intermittent shunting. This helps explain "normal pressure POS", in which patients have normal pulmonary pressures, having profound positional hypoxemia, often reversible with PFO closure [9]. 

Several studies have shown that a PFO can transform a benign anatomical variant into a clinically fatal condition when there is a significant intra atrial shunt. Off-pump coronary artery bypass (OPCAB) is commonly practiced these days. This manipulation of the heart causes disturbed diastolic filling of the right ventricle by direct compression; if there is a PFO, it can lead to fatal hypoxemia; therefore, early detection and closure of PFO can be life-saving in certain conditions [10].

Our case aligns with these reports; her ventilatory requirements, postural symptoms, and normal right-sided pressures suggested a mechanically facilitated shunt rather than intrinsic pulmonary disease. The immediate improvement in oxygenation after device closure further supports the role of the PFO as a major contributor to her refractory hypoxemia. 

## Conclusions

This case shows how a patent foramen ovale (PFO) can become a hidden but reversible cause of persistent hypoxemia. When a patient's presentation is inconsistent with imaging or fails to respond to usual treatment, it is important to look for an intracardiac shunt. Positional changes, unexplained refractory hypoxia, and a mismatch between tests and clinical severity should prompt a bubble study or advanced echocardiography, especially in ventilated patients where bedside clues are limited. PFO closure in such situations may result in a rapid rise in oxygen levels and clinical recovery. 
